# Randomised study within a trial (SWAT) to evaluate personalised versus standard text message prompts for increasing trial participant response to postal questionnaires (PROMPTS)

**DOI:** 10.1186/s13063-021-05452-w

**Published:** 2021-07-28

**Authors:** Lucy Cureton, Ioana R. Marian, Vicki S. Barber, Adwoa Parker, David J. Torgerson, Sally Hopewell

**Affiliations:** 1 Centre for Rehabilitation Research in Oxford, Nuffield Department of Orthopaedics, Rheumatology and Musculoskeletal Sciences, University of Oxford, Oxford, UK; 2 Oxford Clinical Trials Research Unit/Centre for Statistics in Medicine, Nuffield Department of Orthopaedics, Rheumatology and Musculoskeletal Sciences, University of Oxford, Botnar Research Building, Windmill Road, Oxford, OX3 7LD UK; 3 York Trials Unit, University of York, York, UK

**Keywords:** Randomised controlled trial, Trial methodology, Study within a trial, Retention, Postal questionnaire

## Abstract

**Background:**

Use of a person’s name in a text message has been shown to be effective in instigating behaviour change. We evaluated the effectiveness of a personalised text message (including the recipient’s name) versus a standardised text message for prompting a response from trial participants to complete and return postal follow-up questionnaires.

**Methods:**

Using a randomised study within a trial (SWAT) embedded within the host GRASP (Getting it Right: Addressing Shoulder Pain) trial, participants who provided a mobile telephone number were randomised (1:1) by a central computer system to receive either (1) a personalised text message which included their name or (2) a standard text message. Text messages were sent by the trial office on the same day as the 6-month GRASP follow-up questionnaire. The primary outcome was questionnaire response rate, defined as the proportion of 6-month GRASP follow-up questionnaires returned by participants. Secondary outcomes included time to response, the proportion of participants sent a reminder follow-up questionnaire, and cost.

**Results:**

Between March 2017 and May 2019 (recruitment period for GRASP trial), 618 participants were randomised to a personalised (n = 309) or standard (n = 309) text message and all were included in the analysis. The overall questionnaire response rate was 87% (n = 537/618); 90% (n = 277/309) of participants responded in the personalised text message group compared to 84% (n = 260/309) in the standard text message group (relative risk (RR) 1.07; 95% CI 1.00 to 1.13). Participants randomised to receive the personalised text message were more likely to return their initial postal questionnaire than those who received the standard text message (n = 185/309; 60% vs. n = 160/309; 52%) (RR 1.16; 95% CI 1.00 to 1.33); this represents an absolute percentage difference between intervention groups of 8%. Post hoc subgroup analysis showed that males under 65 years were the group most likely to return their initial questionnaire if they received a personalised text message.

**Conclusion:**

Overall, participants who received a personalised text message were more likely to return their questionnaire than those who received the standard text message.

**Trial registration:**

GRASP Trial ISRCTN16539266; SWAT Repository ID 35

**Supplementary Information:**

The online version contains supplementary material available at 10.1186/s13063-021-05452-w.

## Background

Randomised controlled trials are crucial for evaluating healthcare interventions. In undertaking trials, postal self-completed questionnaires are an inexpensive and widely adopted method for collecting patient reported outcomes, especially from large, geographically dispersed populations [1, 2]. However, trialists experience difficulties with maintaining questionnaire response rates from participants, which can introduce bias, reduce the sample size and statistical power, and affect the validity, reliability, and generalisability of findings [1, 3–5].

Short messaging service (SMS) text messaging (‘text messaging’) is a simple, cost-effective, and ubiquitous form of communication. Text messages can be delivered by automated systems, which allow for the content of these messages to be easily and inexpensively varied, so messages can be customised to each recipient. Research on text messages have found them to be effective for instigating behaviour change [6], reducing nonattendance rates for outpatient clinic appointments [7, 8], and for improving recruitment and response rates in trials [9, 10].

The wording of text messages has also been shown to impact on response rates. In a trial to encourage the payment of delinquent fines, using the name of the recipient in the text message was found to be more effective at inducing payment of the fine, than a standardised text message not including the recipient’s name, or even a personalised message with the amount of fine to be paid [11]. Additionally, psychological evidence suggests that the use of a person’s name increases the likelihood of attracting their attention [12], that a person will filter out competing stimuli and refocus their attention when their name is mentioned [13], and that this occurs even when their name appears in printed text [14]. Little research however exists on the use of personalised text messaging for improving trial response rates. A Cochrane systematic review of 38 strategies to improve retention in trials [15] found that while the majority of recruitment interventions focus on postal return of questionnaires, only three trials involved the use of text messages [16–18], and of these, none examined the impact of personalising text messages on response rates or times.

The GRASP trial (Getting it Right: Addressing Shoulder Pain) was a randomised controlled trial assessing the clinical and cost-effectiveness of progressive exercise compared with best practice physiotherapy advice, with or without corticosteroid injection, in adults with a rotator cuff disorder [19]. All of the outcomes were patient reported and participants were asked to complete postal follow-up questionnaires at 8 weeks and 6 and 12 months after randomisation. This follow-up of participants offered an opportunity to evaluate a retention strategy in the trial, with no direct face-to-face follow-up, by conducting a randomised study within a trial (SWAT). The aim of this SWAT was to compare the effectiveness of a personalised text message (including the recipient’s name), versus a standardised text message for prompting trial participants to complete and return postal follow-up questionnaires.

## Methods

### Trial design

The general methodology of this SWAT within the GRASP trial was guided by methodology developed and published by START (Systematic Techniques for Assisting Recruitment to Trials) [20, 21]. Participants were randomised (1:1) to receive one of two interventions: (1) a personalised text message which included their name (intervention group) or (2) a standard text message (control group). Ethical approval was obtained from the Berkshire B Research Ethics Committee (REC) in the form of a substantive amendment to the host trial (REC Ref. 16/SC/0508; Integrated Research Application System (IRAS) ID 199243).

### Participants

All participants in the PROMPTS SWAT were consented and enrolled from the GRASP trial, which acted as the host trial. In addition to meeting the inclusion criteria for the GRASP trial, the following inclusion criteria were applied:

- Participants needed to have the use of a mobile telephone.
- Participants were willing to provide a mobile telephone number and consented for contact to be made by the GRASP trial team using this number.

Messages were sent via a secure third-party text message gateway software (University of Oxford SMS service), so participants were also asked for consent to share their data with this third-party software company to allow the messages to be sent to them. Participants who did not provide a mobile number or did not consent to receive texts were excluded from the PROMPTS SWAT.

### Interventions

Participants were randomised to receive either a personalised text message, which included their name (intervention group) or a standard text message (control group). The text message was sent to trial participants at the same time as their 6-month follow-up postal questionnaire was sent by the trial team; therefore, it would arrive a few days before the participant received their follow-up questionnaire. For anyone (non-responders) who did not return their questionnaire pack at 6 months within 3 weeks of posting out, there then was a cascade of actions that followed:

- A reminder follow-up questionnaire was posted 3 weeks after the initial questionnaire was sent.
- A phone call to the participant was made 5 weeks after the initial questionnaire was sent.
- An email to the participant inviting them to complete the questionnaire online was sent 8 weeks after the initial questionnaire had been posted.

Each text message contained the same core information. Recipients were reminded about the arrival of the questionnaire, about the importance of their responses and to return the postal questionnaire as soon as possible. The wording of the text messages in the intervention and control and groups are outlined in Table 1. For participants in the intervention group, text messages were customised using their name, according to how they preferred to be addressed e.g. Mr Smith, John Smith, or John.

**Table 1:** Messages associated with each intervention in PROMPTS

PROMPTS text message condition	Wording in message
***Control group***	*From the GRASP Trial: We have just sent you a GRASP questionnaire in the post. We would be extremely grateful if when you receive it, you complete it and return it as soon as you can. Thank you*
***Intervention group***	*From the GRASP Trial:* ***[Mr Smith]*** *We have just sent you a GRASP questionnaire in the post. We would be extremely grateful if when you receive it, you complete it and return it as soon as you can. Thank you*

### Outcomes

The primary outcome measure was questionnaire response rate, defined as the proportion of GRASP follow-up questionnaires returned by participants. The secondary outcome measures included time to response (defined as the number of days which elapsed between the GRASP follow-up questionnaire being mailed out to participants and the questionnaire recorded as being returned to the GRASP trial team), the proportion of participants sent a reminder follow-up questionnaire, and the cost of the text message intervention.

### Randomisation and blinding

Participants were assigned a unique trial identification (ID) number by the GRASP trial team. A computer-generated randomisation list was used to list all participants who provided a mobile telephone number. OCTRU’s Registration/Randomisation and Management of Product (RRAMP) web-based system was used to randomise and assign the allocation; the trial administrative team performed all randomisations once eligibility was confirmed. Participants were randomly allocated (1:1) to the intervention and control group. Generation of the allocation sequence and assignment of the intervention and control groups was undertaken independently by a researcher not involved with the delivery of the text messages. To avoid imbalance, block randomisation with equal probabilities of assignment to the intervention and control groups was be used. GRASP trial participants were blinded to the nature and objectives of the PROMPTS SWAT.

### Sample size

As is usual with an embedded trial within a trial, no formal power calculation was undertaken as the sample size was constrained by the number of participants included in the GRASP trial receiving follow-up questionnaires and consenting to use of their mobile telephone number.

### Analysis

All eligible participants were included in the analysis in accordance with the intention-to-treat design. The analyses were conducted in Stata 15.1 (StataCorp). Questionnaire response rates and whether a reminder follow-up questionnaire was sent were compared between using a chi-square test and reported as risk ratios and 95% confidence intervals (CI). The time to return of the questionnaire was plotted using a Kaplan-Meier survival curve and estimated Cox proportional hazard ratios (HR) were calculated for the overall response and those returning the initial questionnaire and the log-rank test used to compare the Personalised and Standard text groups. Observations were censored at 125 days from the time a questionnaire was sent to the participant. The cost of the text message intervention was also calculated. Research staff costs were not calculated as the follow-up of participants was undertaken during the normal time on the host trial. We performed two post hoc subgroup analyses for age (under 65 years/65 years and older) and gender (male/female).

## Results

**Fig. 1:**
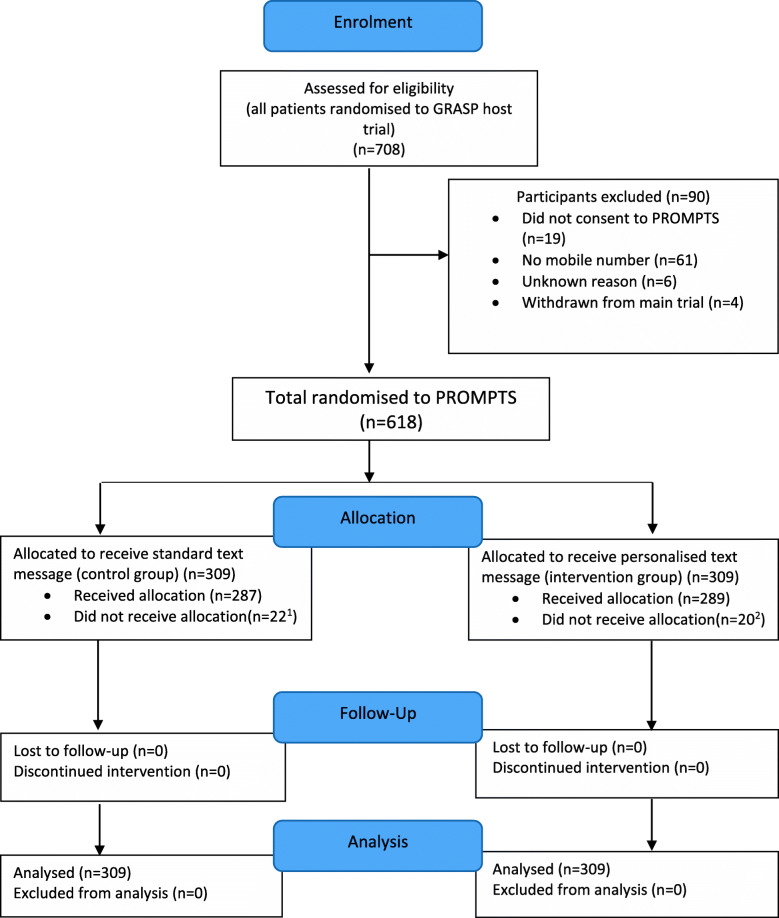
Flow diagram of participants at each stage. ^1^Reasons for not receiving allocation: message not sent in error (*n* = 10), incorrect mobile number (*n* = 5), randomised in error (*n* = 4), and patient withdrawn from host trial (*n* = 3). ^2^Reasons for not receiving allocation: message not sent in error (*n* = 12), incorrect mobile number (*n* = 4), randomised in error (*n* = 2), and patient withdrawn from host trial (*n* = 2). Incorrect mobile number, participant provided 10 not 11 digit mobile telephone number

A total of 708 participants were randomised to the GRASP trial, of which 618 (87%, Fig. 1) were randomised to the PROMPTS SWAT. Three hundred and nine participants were randomised to receive the personalised text message and 309 were randomised to receive the standard text prior to receiving their 6-month follow-up postal questionnaire. Six participants were not sent the 6-month follow-up questionnaire, of these five withdrew from the host trial prior to the follow-up time point, and one was missed from the mail-out and SMS list in error.

The baseline characteristics across both groups were similar. Overall, the mean age was 54 years (standard deviation (SD) 12.7), with the three quarters of participants falling into the under 65 age group (76% under 65 vs. 24% 65 or over) (Table 2). Of the 618 participants randomised into PROMPTS, the overall questionnaire response rate was 87% (*n* = 537); 90% (*n* = 277/309) of participants responded in the personalised text message group compared to 84% (*n* = 260/309) in the standard text message group (risk ratio (RR) 1.07; 95% CI 1.00 to 1.13). This represents an absolute percentage difference between intervention groups of 6% (95% CI 0 to 11%). Seventy-four percent (*n* = 456/537) of participants returned either the original or reminder questionnaire by post, 71% (*n* = 220/309) in the standard text message group, and 76% (*n* = 236/309) in the personalised text message group. Other methods of follow-up included telephone follow-up and online (Table 3). There were 81 participants for which their 6-month questionnaire was classed as ‘missing’ (no data received by any method).

**Table 2:** Baseline characteristics for PROMPTS participants

	Standard text (*N* = 309)	Personalised text (*N* = 309)	Overall (*N* = 618)
**Gender**			
Male	157 (51%)	152 (49%)	309 (50%)
Female	152 (49%)	157 (51%)	309 (50%)
**Age (years)**			
Mean (SD)	54 (12.8)	55 (12.7)	54 (12.7)
**Age group**			
< 65 years	241 (78%)	227 (74%)	468 (76%)
≥ 65 years	68 (22%)	82 (27%)	150 (24%)

*SD* standard deviation

**Table 3:** Overall questionnaire response rate at 6-month time point

	Standard text (*N* = 309)	Personalised text (*N* =309)	Overall (*N* = 618)
Overall response rate*	260 (84%)	277 (90%)	537 (87%)
*Post*	*220 (71%)*	*236 (76%)*	*456 (74%)*
*Telephone*	*39 (13%)*	*33 (11%)*	*72 (12%)*
*Online*	*1 (< 1%)*	*8 (3%)*	*9 (2%)*
*Missing*	*49 (16%)*	*32 (10%)*	*81 (13%)*
Returned initial questionnaire	160 (52%)	185 (60%)	345 (56%)
Returned reminder questionnaire	100 (32%)	92 (30%)	192 (31%)
Did not return questionnaire	46 (15%)	29 (9%)	75 (12%)
Was not sent questionnaire	3 (< 1%)	3 (< 1%)	6 (< 1%)

*Chi-squared test *p* = 0.04

Overall, participants who were randomised to receive the personalised text message were more likely to return their initial postal questionnaire than those who received the standard text message (*n* = 185/309; 60% vs. *n* = 160/309; 52%) (RR 1.16; 95% CI 1.00 to 1.33); this represents an absolute percentage difference between intervention groups of 8% (95% CI 0 to 16%) (Table 3). Conversely, participants who were randomised to receive the personalised text message were less likely to need to be sent a reminder postal questionnaire than those who received the standard text message (*n* = 121/309; 39% vs. *n* = 146/309; 47%) (RR 0.83; 95% CI 0.69 to 0.99) (Table 3).

**Fig. 2:**
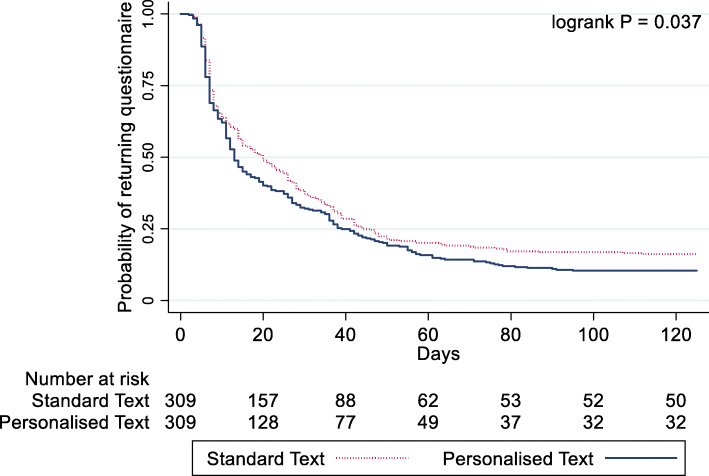
Kaplan-Meier curves and log-rank test of time to overall response personalised and standard text messages (*n* = 618)

**Fig. 3:**
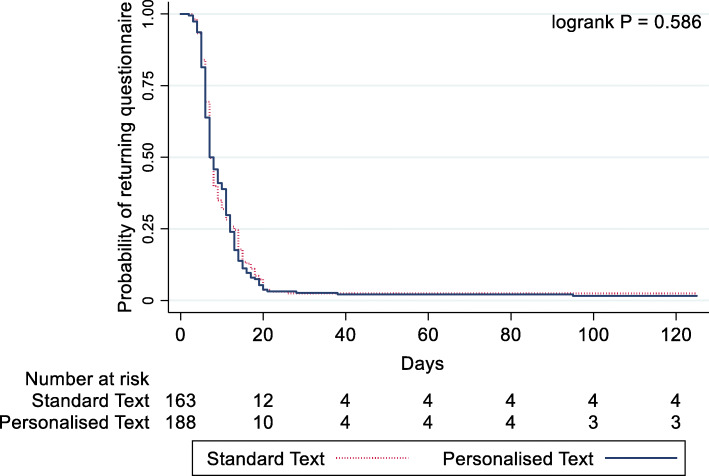
Kaplan-Meier curves and log-rank test of time to response for initial questionnaire for personalised and standard text messages (*n* = 344)

The time taken to return the questionnaire regardless of whether a reminder was sent or not (*n* = 537/618) ranged between 2 and 125 days (median 13, IQR 7 to 30). This was slightly lower (median 12, IQR 7 to 29) in the personalised text group compared with those who received the standard text (median 14, IQR 7 to 31) (Table 4). An estimated HR of questionnaire return for the standardised group relative to personalised text was 1.19 (95% CI 1.00 to 1.41, *p* = 0.043) when accounting for censored data. A Kaplan-Meier plot for the two groups and their associated time to response is presented in Fig. 2. The time taken to return the initial questionnaire (*n* = 344/618) ranged between 2 and 95 days (median 7, IQR 6 to 12). This was similar (median 7, IQR 6 to 12) in the personalised and standard text message group (median 7, IQR 6 to 12) (Table 4) (HR 1.06 95% CI 0.85 to 1.31, *p* = 0.6 19; Fig. 3).

**Table 4:** Time to response overall and initial questionnaire by randomised group and overall

	Standard text (*N* = 309)	Personalised text (*N* = 309)	Overall (*N* = 618)
Overall response rate	260 (84%)	277 (90%)	537 (87%)
Time to response (days)			
*Range (min to max)*	*3 to 125*	*2 to 95*	*2 to 125*
*Median (IQR)*	*14 (7, 31)*	*12 (7, 29)*	*13 (7, 30)*
Returned initial questionnaire	159 (52%)	185 (60%)	344 (56%)
Time to initial response (days)			
*Range (min to max)*	*3 to 26*	*2 to 95*	*2 to 95*
*Median (IQR)*	*7 (6 to 12)*	*7 (6 to 12)*	*7 (6 to 12)*

Two post hoc subgroup analyses were performed for age group and gender. The data suggest that participants aged under 65 years old were more likely to return their initial questionnaire if they received a personalised text message (*n* = 124/227; 55%) than those under 65 who received the standard text message (*n* = 111/241; 46%) (Table 5). Overall, participants aged 65 and older were more likely to return their initial questionnaire with little or no difference between the standard (*n* = 49/68; 72%) and personalised (*n* = 61/82; 74%) text message. The number of males and females returning the initial questionnaires was very similar; however, males seemed more likely to respond positively to a personalised reminder text message (*n* = 94/152; 62%) compared to a standard text message (*n* = 78/157; 50%).

**Table 5:** Initial questionnaire response rate based on age and gender

	Standard text (N = 309)	Personalised text (N = 309)	Overall (N = 618)
Returned initial questionnaire	160/309 (52%)	185/309 (60%)	345/618 (56%)
< 65 years	111/241 (46%)	124/227 (55%)	235/468 (50%)
≥ 65 years	49/68 (72%)	61/82 (74%)	110/150 (73%)
Male	78/157 (50%)	94/152 (62%)	172/309 (56%)
Female	82/152 (54%)	91/157 (58%)	160/309 (56%)

The cost of the text message itself was very inexpensive (each SMS text message cost £0.08 per message, a total cost of £49.44 for all messages sent). However, there were associated activities involved in sending SMS messages. Most of these activities were related to implementation of the SWAT within the host trial and/or could potentially be automated (e.g. randomisation of each patient into the SWAT, cross-checking and inputting patient name and mobile number, sending each SMS, logging message sent). Sending the personalised text message involved looking up their name and manually inserting it into the SMS, so it took slightly longer than sending the standard text message.

## Discussion

In this SWAT, embedded within a host trial of adults with shoulder pain, participants randomised to receive the personalised text message were more likely to return a questionnaire (either initial or reminder) compared with those receiving the standard text message. In addition, participants receiving a personalised message were less likely to need a reminder questionnaire than those receiving the standard text message.

Since publication of the Cochrane review of strategies [15] to improve retention in randomised trials, several other studies have evaluated the effects of an electronic prompt compared to no electronic prompt on questionnaire response rates, with differing results and in different trial populations. In a SWAT involving people with chronic obstructive pulmonary disease, an electronic prompt (either text message or email) increased questionnaire response rates compared to no electronic prompt (70% vs. 61%; difference 9%; 95% CI − < 1 to 18%). The mean time to response was 23 days in the electronic prompt group and 33 days in the control group (HR = 1.27; 95% CI 1.105 to 1.47). The results were combined with two previous studies in a meta-analysis, which showed an increase in response rate of 7% (95% CI < 1 to 13%) [9]. Conversely, several subsequent SWATs comparing text message pre-notification versus no pre-notification prior to sending out postal questionnaires found no different in retention rates; one involved parents of babies with eczema [22], one was in adults with kidney stones [23], and one involving adults with depression [24].

**Fig. 4:**
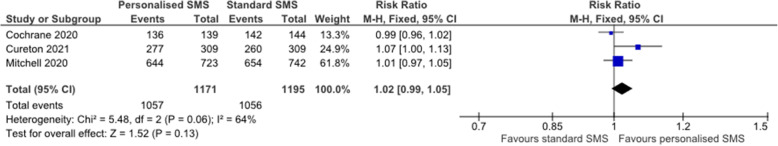
Personalised versus standard text message: overall questionnaire response rate

**Fig. 5:**
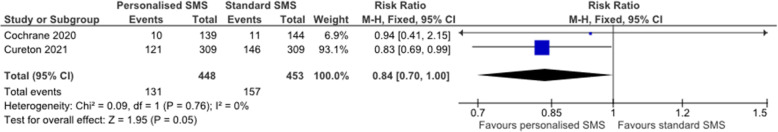
Personalised versus standard text message: reminder questionnaire sent

We are aware of two studies specifically comparing the effects of a personalised versus standard text message on questionnaire response rates. One SWAT was embedded within a trial of falls prevention in older adults [25] and another involved adults undergoing total knee joint replacement surgery [26]. Combining the results of these two previous studies, with our results of our SWAT showed no difference between a personalised and standard text message in overall response questionnaire rate (RR 1.02; 95% CI 0.99 to 1.05; 3 studies; 2366 participants) (Fig. 4). However, there was a small reduction in the number of people who needed to be sent a reminder postal questionnaire in those who received a personalised text message (RR 0.84; 95% CI 0.70 to 1.00; 2 studies; 901 participants) (Fig. 5).

Sending trial participants a text message the same time as their 6-month follow-up postal questionnaire took little time to do, especially compared to the time and effort taken to print, prepare, and send out paper questionnaires. The ‘instant’ nature of a text message was reassuring; sending postal questionnaires invariably comes with a time lag, so it was a good way of knowing that a participant had been reminded of the host trial in real-time. The SMS system retained a log of all messages that were sent by the team, so it was easy to check that messages had been sent out as planned. The messages could be queued up to be sent at specified dates and times, so in the event of the team having annual leave, messages could be prepared and queued up beforehand. The time taken to send the messages, combined with how inexpensive they were to send, made the study very worthwhile to do, as even a small improvement in retention was worth the expended effort.

One limitation of our SWAT was that the research teams at sites were responsible for noting down the participant’s mobile numbers. In a busy hospital environment, it is easy to type in a mobile number incorrectly. The SMS service we used was limited in that it did not identify if a text message had gone to an incorrect number, so this did not give an opportunity for the team to attempt to obtain the correct number from the participant or site. We were only able to identify an incorrect mobile number for nine participants, as it did not include the required number of digits (i.e. 10 digits rather than 11). In addition, the SMS system used to implement this was SWAT was not automated which meant that the trial team had to look up each participants name and mobile number from the trial database and then populate the message field, making sure that the correct template was selected. A more sophisticated text message service would enable this process to be automated and therefore saving time and cost if implemented on a larger scale.

In our SWAT, post hoc subgroup analysis showed that males under 65 years were the group most likely to return their initial questionnaire if they received a personalised text message. It would be important for future research in this area to look at the effectiveness of an SMS text message on retention in different age groups, by gender and in different disease settings. Different media platforms or mobile apps should also be considered, as SMS text messaging might become less popular in the future, especially in younger age groups. In this SWAT, the text message was sent to the participant at the same time that the questionnaire was put in the post. Further research could look at whether the timing of the text message makes the intervention more or less effective, for example, sending the text message at the same time that the participant is likely to receive the questionnaire.

## Conclusion

Overall, participants who received a personalised text message were more likely to return their questionnaire (either initial or reminder) than those who received the standard text message. Future trials should consider implementing text message reminders, as this is a cost-effective and time-saving way of streamlining the postal questionnaire process and improving retention. As sending text messages is relatively simple and easy, this method could be applied to a wide range of different trial designs and participant populations.

## Supplementary Information

**Additional file 1.** CONSORT 2010 checklist of information to include when reporting a randomised trial

## Data Availability

The dataset used and analysed during the SWAT are available from the corresponding author on reasonable request.
